# Correction to: Targeted metabolomics analyses for brain tumor margin assessment during surgery

**DOI:** 10.1093/bioinformatics/btad009

**Published:** 2023-01-16

This is a correction to: Targeted metabolomics analyses for brain tumor margin assessment during surgery, *Bioinformatics*, Volume 38, Issue 12, 15 June 2022, Pages 3238–3244, https://doi.org/10.1093/bioinformatics/btac309

In the originally published version of this manuscript, there was an error in the online record of the affiliation for the 2^nd^ author, Dr Kaynar. This should read “Computer Engineering Department, Bilkent University, Ankara 06800, Turkey” instead of “School of Computer Science, McGill University, Montreal, QC H3A 0E9, Canada”.

This error has been corrected online.

